# America’s Most Distressed Areas and Their Neglected Infections: The United States Gulf Coast and the District of Columbia

**DOI:** 10.1371/journal.pntd.0000843

**Published:** 2011-03-29

**Authors:** Peter J. Hotez

**Affiliations:** 1 Sabin Vaccine Institute, Washington, D.C., United States of America; 2 Department of Microbiology, Immunology, and Tropical Medicine, George Washington University, Washington, D.C., United States of America

The neglected infections of poverty represent the latest threat to the poorest people living on the Gulf Coast of the United States and in Washington, District of Columbia.

Together, Hurricanes Katrina and Rita and the BP oil disaster have shed light on a tragic level of poverty in the northern Gulf of Mexico, especially for the African Americans living in Louisiana, Mississippi, and Alabama. According to the National Center for Children in Poverty at Columbia University, more than 40% of black children in each of these states currently live in poor families, and over 12% of children from Louisiana and Mississippi live in extreme poverty, defined as families with incomes that are less than half of the federal poverty level [1]. Overall, the 12 million people living in Louisiana, Mississippi, and Alabama suffer from the lowest incomes and educational attainment, as well as the shortest life expectancy, compared to anywhere else in the United States [2]. The finding that combined these Gulf Coast states have the nation’s lowest human development index scores has prompted a call by the American Human Development Project to launch a Marshall Plan for the Gulf, referring to a comprehensive reconstruction plan that resembles US efforts to reconstruct Europe in the devastation that followed World War II [2].

In previous papers I have noted high rates of parasitic and related neglected infections among the poorest Americans living in distressed areas, but especially in inner cities, the American South, the border with Mexico, and Appalachia [3]–[5]. Indeed, the rates of some of these neglected infections of poverty among African Americans are comparable to the rates found in Nigeria [5]. These and other findings led to the 2009 National Summit on Neglected Infections of Poverty in the United States held in Washington, D.C. [6]. The summit coincided with new bipartisan legislation introduced in the US House of Representatives by Representative Hank Johnson (Georgia) asking for a report from the Department of Health and Human Services Secretary on the full spectrum of the neglected infections of poverty in America’s distressed areas, with emphasis on enhanced surveillance and efforts to determine the level of autochthonous transmission, i.e., transmission occurring within US borders [7].

**Figure 1:**
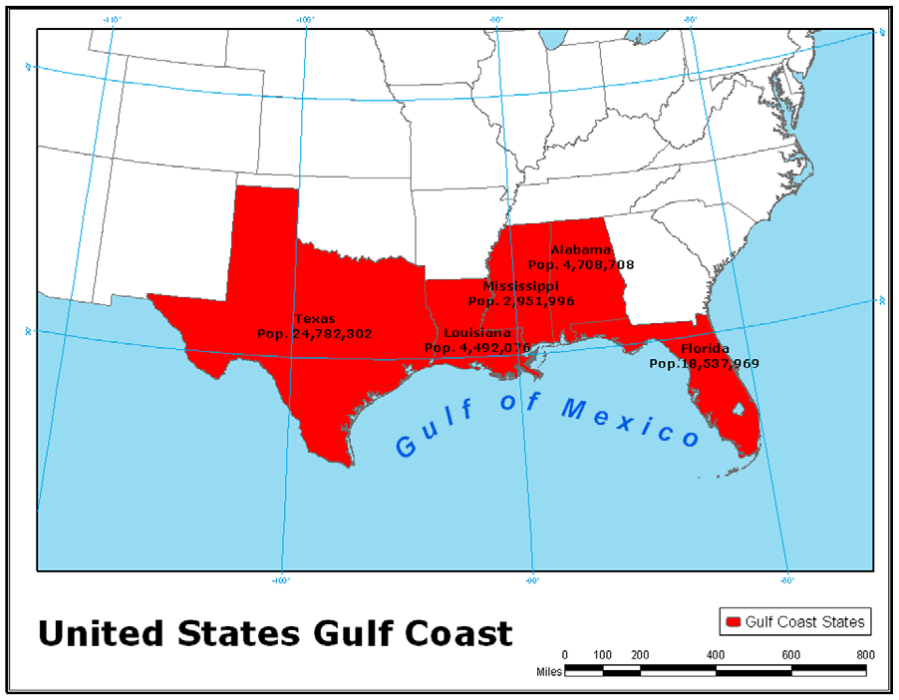
Map of the United States Gulf Coast. Source: Map created by Jessica Schwartz, The George Washington University; population data obtained from [32].

A fresh look at the neglected infections of poverty reveals the extreme vulnerability of the US Gulf Coast to these conditions, which includes Louisiana, Mississippi, and Alabama, as well as neighboring regions of Texas and Florida (Figure 1). Among the vector-borne neglected infections, there is now evidence of Chagas disease transmission in Louisiana, including a rural area within the borders of New Orleans [8], [9], and the emergence of cutaneous leishmaniasis in South Texas [10]. Among the most important factors responsible for the emergence of these diseases is extreme poverty associated with poor housing without plumbing, air conditioning, or window screens [8], [11].

Since I first wrote about neglected infections in the US two years ago, the prospect of dengue and its most serious complication, dengue hemorrhagic fever, have emerged as additional and now imminent threats to the people living on the Gulf Coast. Between 1946 and 1980, no cases of dengue occurred in the continental US [12]. Beginning in 1981, however, dengue hemorrhagic fever first appeared in the Caribbean [13], and almost 5 million cases of dengue were reported in the Caribbean and Central America between 2000 and 2007 [12], [14]. During this period, dengue virus type 2 (DENV-2) and dengue hemorrhagic fever appeared in the Gulf coast cities of Matamoros and Brownsville in Mexico and Texas, respectively [15], [16]. The significantly higher dengue rates on the Mexican side were associated with higher levels of poverty and poor sanitation and housing, which promote breeding habitats of *Aedes* mosquitoes [16]. In 2009, DENV-1 appeared suddenly in the Florida Keys, representing the first dengue case acquired locally in Florida since 1934 [12]. Together with widespread dengue in the Caribbean and Central America, the emergence of dengue on the Gulf coasts of Florida and Texas could portend widespread dengue outbreaks, including the possibility of dengue hemorrhagic fever, in areas of extreme poverty along the northern Gulf of Mexico.

Non-vector-borne neglected infections are also now highly prevalent among the poorest people living on the Gulf Coast. Trichomoniasis exhibits a 10-fold higher prevalence among African American women [17], and rates of this parasitic infection are particularly high among women who attend outpatient HIV and family planning clinics in New Orleans, Louisiana [18]–[21]. Trichomoniasis was shown to be an important co-factor in the HIV/AIDS epidemic in New Orleans and presumably elsewhere on the Gulf Coast, where it is associated with increased HIV shedding [19], [20], and there is additional concern that metronidazole drug resistance strains of this parasitic protozoan have emerged in New Orleans [19]. When they were last examined during the 1970s, ascariasis and other intestinal parasitic helminth infections were found to be highly prevalent in southern Louisiana (Lafayette, Baton Rouge, and Independence, Louisiana), especially among African Americans [22], [23], but these neglected diseases have not been studied since [3]. Toxocariasis, a related helminth infection transmitted from dogs, is likely the most common helminth infection in the US [3], with a prevalence among African Americans that exceeds 20%, especially in the South [24]. There are no recent toxocariasis prevalence estimates reported for the Gulf Coast states, although *Toxocara* eggs have been detected in soil samples in Baton Rouge [25]. *Toxocara* was recently noted to frequently occur with toxoplasmosis co-infections [26]. Both toxoplasmosis and cytomegalovirus (CMV) infection are important congenital infections that disproportionately affect African American populations and are associated with severe neurological deficits [27], [28].

Although the District of Columbia does not have statehood, as a unique federal district it is often treated as an autonomous region and compared in rankings with the 50 US states. Today, Washington, D.C., rivals Louisiana, Mississippi, and Alabama as among the worst in terms of life expectancy and health index [2]; with respect to poverty indices, a recent report entitled “DC’s Two Economies” revealed that in terms of employment status, income, and poverty levels, Washington, D.C., currently exhibits some of the greatest disparities between whites and blacks of any city in the US [29]. An astonishing 6.5% of African American males in the District of Columbia are also HIV positive [30] (Table 1). Data for neglected infections of poverty in Washington, D.C., are practically non-existent, although some information from neighboring Baltimore indicates that trichomoniasis is extremely common [31]. Thus, we urgently need a program for active surveillance for the most common neglected infections of poverty in the District of Columbia.

**Table 1:** Rates of Persons Living with HIV/AIDS in Washington, D.C., by Ward.

DC Ward	Population (2000)	Rates of Persons Living with HIV/AIDS* (2007, % Population)	% Population Black, Non-Hispanic (2000)	% Population White, Non-Hispanic (2000)	% Population Hispanic (2000)	Poverty Rate (2000, %)
1	73,334	2.50	46.0	25.0	25.0	22.0
2	68,827	2.10	20.0	61.0	10.0	19.0
3	73,753	0.30	6.2	80.0	6.6	7.5
4	75,001	1.70	71.0	15.0	12.0	12.0
5	71,604	2.70	88.0	7.4	3.0	20.0
6	68,087	2.80	63.0	30.0	3.2	21.0
7	70,539	2.40	97.0	1.2	0.8	25.0
8	70,915	2.80	93.0	5.1	1.3	36.0

Rates are among adults and adolescents only in the population. Sources: HIV/AIDS data [30]; population and poverty data [33].

If reconstruction plans for the Gulf Coast and Washington, D.C., are ever implemented, an important component should be directed towards the neglected infections of poverty in these areas. I am particularly concerned about the Gulf’s vulnerability to emerging dengue fever infections, while for both the Gulf Coast and Washington, D.C., we must urgently address neglected diseases that disproportionately affect African American populations, including toxocariasis, toxoplasmosis, trichomoniasis, and congenital CMV infection; and those that affect Hispanic Americans, including Chagas disease and cysticercosis. The fact that we know so little about the neglected infections of poverty in America’s most distressed areas is representative of just how glaring these conditions are as health disparities.

In 2008, I suggested that we must urgently address the neglected infections through programs of active surveillance and assessments of disease burdens, studies to examine the mechanisms of transmission in poor communities, efforts to control or eliminate these infections through public health interventions that include treatment or vaccination and health education, and a program of research and development to create a new generation of drugs, diagnostics, and vaccines [3]. Since then, little has changed except for the National Summit and the re-introduction of Rep. Johnson’s bill to the House.
